# Global lessons from local contexts: The evolution of biomedicine education in Spain

**DOI:** 10.1371/journal.pone.0335051

**Published:** 2025-10-27

**Authors:** Francisco J. Muñoz, Esther Esteban, Alberto Pérez-Mediavilla, Luis Capitán

**Affiliations:** 1 Departament de Medicina i Ciències de la Vida, Facultat de Medicina i Ciències de la Vida, Universitat Pompeu Fabra, Barcelona, Spain; 2 Departament de Biologia Evolutiva, Ecologia i Cièncis Ambientals, Facultat de Biologia, Universitat de Barcelona, Barcelona, Spain; 3 Departamento de Bioquímica y Genética, Facultad de Ciencias, Universidad de Navarra, Navarra, Spain; 4 Departamento de Cirugía, Facultad de Medicina, Universidad de Sevilla, Sevilla, Spain; Endeavour College of Natural Health, AUSTRALIA

## Abstract

Driven by the presence of faculty with research and clinical backgrounds, and by labor market trends favoring applied training, Biomedicine has emerged as a growing academic field in Spain. This study provides a descriptive analysis of undergraduate Biomedicine programs offered by 18 Spanish universities since 1998, focusing on structural, academic, and outcome-related variables. Data indicate a progressive increase in program availability and student enrollment over the past two decades, reaching a total of 4,614 students in the most recent academic period. Admission criteria remain highly selective, with a mean entry score of 12.5 out of 14. In the absence of guidelines, the curricula from the different universities show a consistent structure, with an emphasis on foundational biomedical sciences in the early academic years – such as Cell Biology, Biochemistry, and Immunology – and the incorporation of advanced subjects in later stages, including Cancer Biology and Bioinformatics. These programs frequently incorporate practical components and research exposure. Over 100 active international collaboration agreements were identified across the institutions studied, reflecting efforts to internationalize their Biomedicine programs. Despite heterogeneity in curricular design, the average graduation rate for the 2022–2023 academic year was 81.8%, and employment outcomes averaged 82.9% over the past decade. The findings suggest a convergence of academic, professional, and institutional factors shaping the development of Biomedicine education in Spain.

## Introduction

Biomedicine degree programs are centered on understanding human health and disease, advancing medical research, and translating discoveries into clinical practice. These programs emphasize interdisciplinary collaboration, public health, and preventive medicine, reflecting a global commitment to addressing complex health challenges [1]. Internationally, biomedical research is recognized for its profound contributions to understanding disease mechanisms, from molecular interactions to integrated human systems [1,2], and for developing innovative diagnostic and therapeutic approaches [3]. Training in this field prioritizes exposure to diverse disciplines and cutting-edge technologies, fostering advancements that bridge science and medicine [4]. Importantly, biomedical sciences and medicine are distinct yet complementary: while biomedicine focuses on laboratory research and population-level disease understanding, medicine applies this knowledge to diagnose, treat, and prevent diseases at the individual level. This synergy underscores the unique value of biomedicine education.

Our study investigates the development of undergraduate Biomedicine programs in Spain, which were established within Faculties of Health and Biological Sciences through collaborations between basic researchers and clinicians. We examine how these programs have expanded across 18 universities since 1998, analyzing their curriculum design, admission competitiveness, and alignment with global biomedical education trends. The study specifically addresses whether this organic, institution-driven growth has responded to labor market demands and prepared graduates for research and healthcare careers, while maintaining academic outcomes through periods of educational disruption like the COVID-19 pandemic.

## Methods

### Participating institutions

Eighteen universities participated in this study, providing a robust sample that spans a diverse array of institutions and regional contexts. The nine public universities are Universidad de Alcalá (Degree in Biología Sanitaria), Universitat Autònoma de Barcelona (Degree in Ciencias Biomédicas), Universitat de Barcelona (Degree in Ciencias Biomédicas), Universidad de Cantabria (Degree in Ciencias Biomédicas), Universitat de Lleida (Degree in Ciencias Biomédicas), Universitat Pompeu Fabra (Degree in Biología Humana), Universidad de Sevilla (Degree in Biomedicina Básica y Experimental), Universitat de Universidad de València (Degree in Bioquímica y Ciencias Biomédicas) and Universidad de Valladolid (Degree in Biomedicina y Terapias avanzadas). The nine private universities are Universidad Alfonso X el Sabio (Degree in Biomedicina), Universidad Camilo José Cela (Degree in Biomedicina), Universidad Europea de Madrid (Degree in Biomedicina), Universidad Francisco de Vitoria (Degree in Biomedicina), Universitat Internacional de Catalunya (Degree in Ciencias Biomédicas), Universidad de Navarra (Degree in Bioquímica), Universidad Ramon Llull (Degree in Biomedicina), Universidad de San Jorge (Degree in Biomedicina) and Universitat de Vic (Degree in Biomedicina). All the universities participating in this study have surpassed the quality criteria determined by the national agency of quality (ANECA) or by the regional agencies (AQU in Catalonia).

### Data collection

Data were obtained from publicly available sources provided by each university (S1 Table) and supplemented by a structured survey sent to the deans, vice-deans, and degree coordinators of Biomedicine programs at 18 Spanish universities, all members of AND Biomed (Spanish National Association of Deans of Biomedicine). Respondents were asked to send us data already available on their institutional websites in order to facilitate and expedite data collection. Specifically, the requested information included: university name, faculty, degree name, year of implementation, admission grade marks per academic year, total number of students per academic year, percentage of international students per academic year, number of national and international institutions participating in mobility programs per academic year, graduation rate per academic year, and employment rate 1–4 years after graduation. All the data provided via the survey are publicly accessible in the university’s official web pages (S1 Table). No personal or identifiable information was collected, and all data used in this study are anonymous and aggregated at the institutional level. The differences in the time spans among the figures are due to the limited availability of data, particularly in the earlier years, as most universities began offering Biomedical Science degree programs after 2008.

This study is a descriptive analysis of academic programs in Biomedicine (university offerings, curricula, graduation rates, international agreements, employability, etc.). It relied exclusively on anonymized and aggregated data derived from institutional-level metrics and were either directly extracted from official university websites (S1 Table) or provided by administrative staff in response to a standardized data request. No personal, sensitive, or identifiable information was collected at any stage of the study.

In accordance with international guidelines – including the EU GDPR (Recital 26), the U.S. Common Rule (45 CFR 46.104), and the Declaration of Helsinki (2013) – research based solely on anonymized, aggregated, and publicly available data does not require ethical review or informed consent. Consistent with this principle, the Institutional Committee for Ethical Review of Projects of University Pompeu Fabra (CIRED-UPF) confirmed that Ethics Committee approval was not required for this study.

### Statistical analysis

The collected data are specifically from the Biomedicine programs of each university. The data are expressed as the mean ± standard error of the mean (SEM), with the number of programs indicated in parentheses in each graph. SEM was used to represent variability in the figures because each data point corresponds to an aggregated mean for a given academic year. SEM indicates the precision of these mean values, which is appropriate for comparing trends over time using linear regression. To analyze the trend over the academic years, we performed a linear regression analysis. This involved fitting a straight line (trend line in blue in all the graphs) to elucidate if there is a linear relationship between the time and the measured values. The goodness of fit of this trend line was assessed using the coefficient of determination (R²).

## Results

### Evolution of biomedicine education in Spain: Growth, student demographics, and academic trends

**Fig 1:**
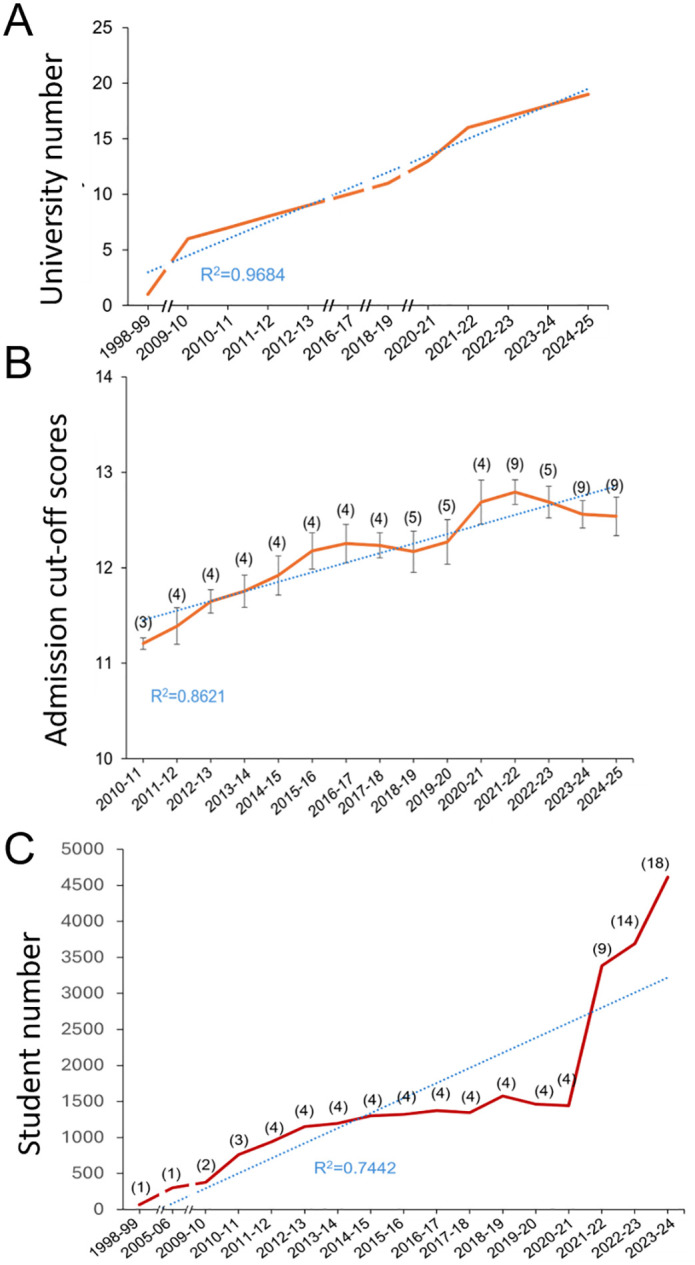
Evolution of Biomedicine Education in Spain: Growth, Student Demographics, and Academic Trends. **A.** Number of Spanish universities including biomedicine degrees in their academic offering. Data shows the number of universities that have biomedicine degrees from the 1998−99 academic year to the 2024−25. Breaks indicate that data is unavailable for the academic years not represented in the graph. **B.** Entry marks. Data shows the mean ± SEM of the admission cut-off scores for the number of public universities indicated in parentheses in the last fifteen academic years. The other 9 private universities have different admission processes and are not included in this data. **C.** Number of students in biomedicine. Data shows the total number of students enrolled per academic year from 1998−99 until the present year. The number of universities is indicated in parentheses. Breaks indicate that data is unavailable for the academic years not represented in the graph.

Since its inception in the 1998−99 academic year, the number of universities offering biomedicine degrees has expanded, reaching 18 institutions in the 2024−25 academic year (Fig 1A). This growth has followed a consistent upward trajectory, with the number of universities doubling every 10 years until 2019 and accelerating in the past five years (R² = 0.9684).

A pivotal moment occurred in 2007, when Spain aligned its university degrees with the European Higher Education Area (EHEA), also known as the Bologna Plan, leading to a redesign of academic priorities and curricula, including those for medicine and biomedicine [5]. From 2009 onward, biomedicine programs experienced substantial growth, with the number of universities offering the degree rising to six by 2009 and reaching nine by 2012. By 2016, ten universities had established biomedicine programs. A pronounced surge occurred in 2020, with the number of institutions increasing to thirteen, likely influenced by global health challenges such as the COVID-19 pandemic [6]. Today, biomedicine degrees are offered by 18 universities, representing approximately 19.8% of Spain’s 91 universities (50 public and 41 private) [7]. Among public universities, 18% offer biomedicine degrees, while private institutions show a slightly higher proportion at 22%.

Admission to biomedicine programs in Spain is highly competitive. Entry marks, calculated from high school grades (60%) and national or regional university entrance exams (40%), have consistently remained high over the past 15 years (R² = 0.8621) (Fig 1B). The average entry mark, measured on a 14-point scale, indicates the academic standards.

Data show that student enrollment in biomedicine programs has also seen significant growth. From 2011−12–2020−21, data from four institutions revealed a steady increase in enrollment (R² = 0.7442) (Fig 1C). By 2023−24, the total number of students enrolled in biomedicine programs reached 4,614 across the 18 participating universities.

### Degree program curricula and learning objectives

**Fig 2:**
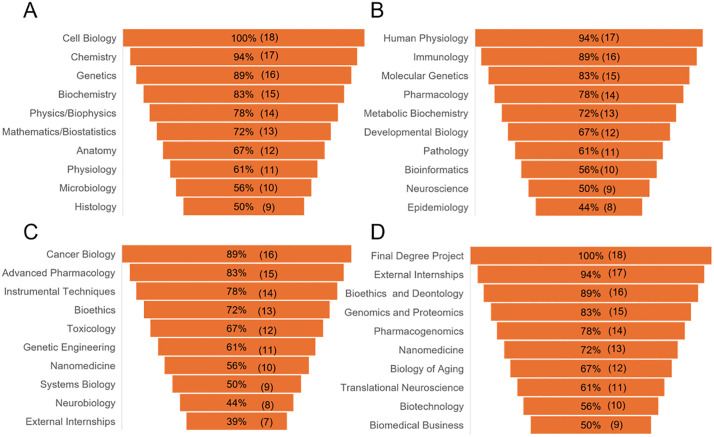
Common subjects in biomedicine degree curricula. Data shows the subjects in the first year **(A)**, second year **(B)**, third year (**C**) and fourth year **(D)**. The data for each subject shown in the stacked diagram indicates the percentage of universities offering that subject within the course, relative to the total number of universities, which is considered 100%, and in parentheses, the universities that include the subject in that course.

The biomedicine curricula of the 18 universities analyzed in this study, as published on their official websites (S1 Table), share a broadly similar structure (Fig 2). Programs typically begin with foundational subjects in the first year, such as Cell Biology, Chemistry, Genetics, Biochemistry, and Physics/Biophysics. These courses establish core scientific knowledge at the cellular and molecular levels.

In the second year, the results show that the focus shifts to more advanced topics, including Human Physiology, Immunology, Molecular Genetics, Metabolic Biochemistry, and Pathology. These courses build on earlier material and introduce mechanisms related to health and disease.

The third year aims specialized knowledge and practical skills, with subjects such as Cancer Biology, Pharmacology, Toxicology, Instrumental Techniques, and Genetic Engineering. External internships are introduced at this stage to provide applied experience.

In the fourth year, students complete a Final Degree Project and continue with internships. The curriculum includes advanced courses such as Pharmacogenomics, Genomics and Proteomics, and Translational Neuroscience.

Beyond the core curriculum, optional subjects (over 25 options) allow students to tailor their education to their interests. Results show that the most common optional courses include Biomedical Engineering (30%, 5 universities), Epigenetics (25%, 4 universities), and Nanomedicine (25%, 4 universities), highlighting a focus on technology-driven and molecular approaches. Other prevalent options are Cancer Biology (20%, 3 universities), Human Genetics (20%, 3 universities), Synthetic Biology (20%, 3 universities), Nutrition (20%, 3 universities), and Bioinformatics (20%, 3 universities).

On the other hand, all curricula prioritize learning objectives that balance content knowledge, practical skills, and professional competencies as they indicate in their respective web pages. These objectives can be summarized as follows: i) Educating students in the fundamental principles of biomedical sciences; ii) Equipping students with instrumental techniques, data analysis, and computational tools for high-quality research; iii) Training students in generic competencies, such as critical thinking, professional practice, and ethical responsibility; iv) Preparing students to apply their knowledge and skills in professional biomedical contexts.

### Internationalization

**Fig 3:**
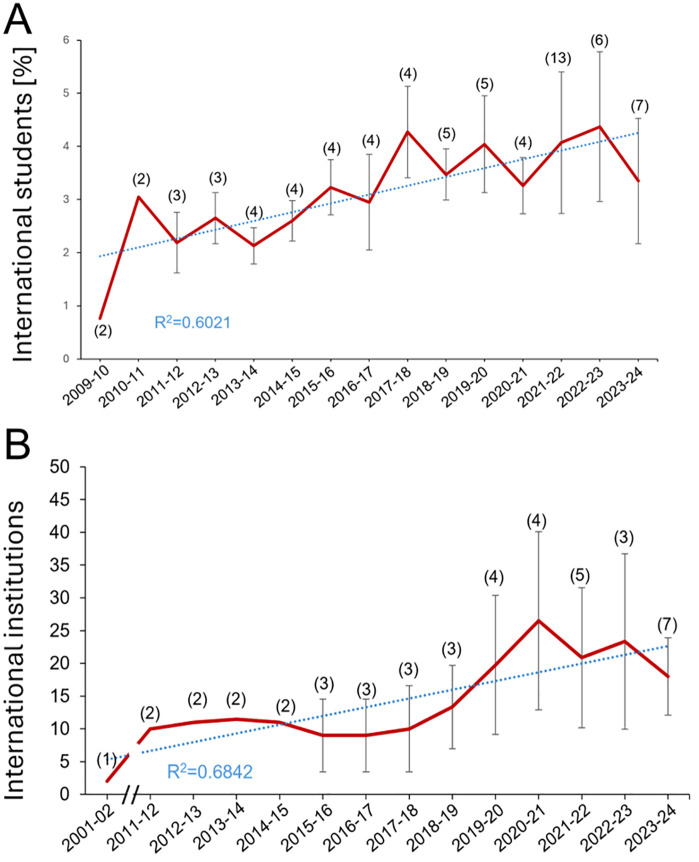
Internationalization of biomedicine studies. **A.** International students in Spanish biomedicine degree. Data shows the mean ± SEM of the percentages of international students regarding the total of students (national and international) in the Spanish universities (number of universities indicated in parentheses) from 2009−10 until the present year. The duration of stay at Spanish universities goes from 3 months to 1 academic year, mostly linked to the Erasmus programs. **B.** Agreements with international institutions. Data shows the mean ± SEM of international institutions that have agreements with Spanish universities allowing the mobility of biomedicine students from 2001−02 until the present year. The number of Spanish universities is indicated in parentheses. Breaks indicate that data is unavailable for the academic years not represented in the graph.

Data on the percentage of international students in biomedicine programs across Spanish universities reveal a trend toward internationalization (R² = 0.6021) (Fig 3A). While fluctuations are observed, the overall increase in international student enrollment suggests a growing appeal of Spanish biomedicine programs on the global stage. Most international students participate through the Erasmus program, with stays ranging from 3 months to 1 academic year [8].

Similarly, the number of international agreements signed by Spanish universities offering biomedicine degrees has shown significant growth (R² = 0.6842) (Fig 3B). In the 2023−24 academic year, a total of 115 agreements were established with institutions across Europe, including countries such as Austria, Belgium, Czech Republic, Denmark, Finland, France, Germany, Greece, Hungary, Iceland, Ireland, Italy, Lithuania, Netherlands, Norway, Portugal, Slovakia, Sweden, Türkiye, and the United Kingdom.

However, the variability in the number of agreements reflects the dynamic nature of international partnerships, as most agreements are renewed annually. Additionally, gaps in data reporting by some universities contribute to this variability.

### Graduation rates

**Fig 4:**
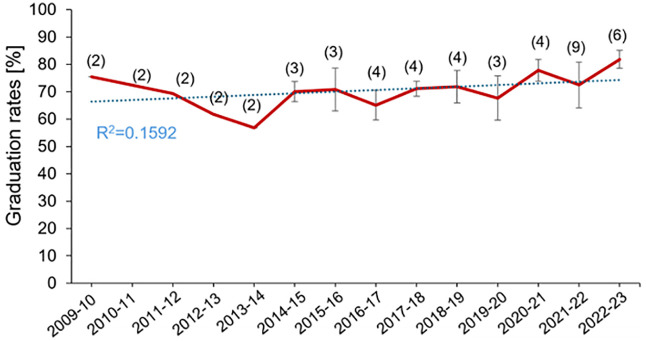
Graduation rates. Data shows the mean ± SEM of the graduation rates in biomedicine degrees from 2009−10 until 2022−23. The number of Spanish universities is indicated in parentheses.

The graduation rates for Biomedicine degrees in Spain have significantly been high over the years (Fig 4). The graduation rate has remained high from 2014–15 to the present. There is no significant effect of the COVID-19 pandemic on graduation rates despite it having been reported that the pandemic seriously affected student engagement [9]. The graduation rate stabilized around 81.90% in 2023–2024 (data from only 4 universities), reflecting a consistent performance.

### Employability

**Fig 5:**
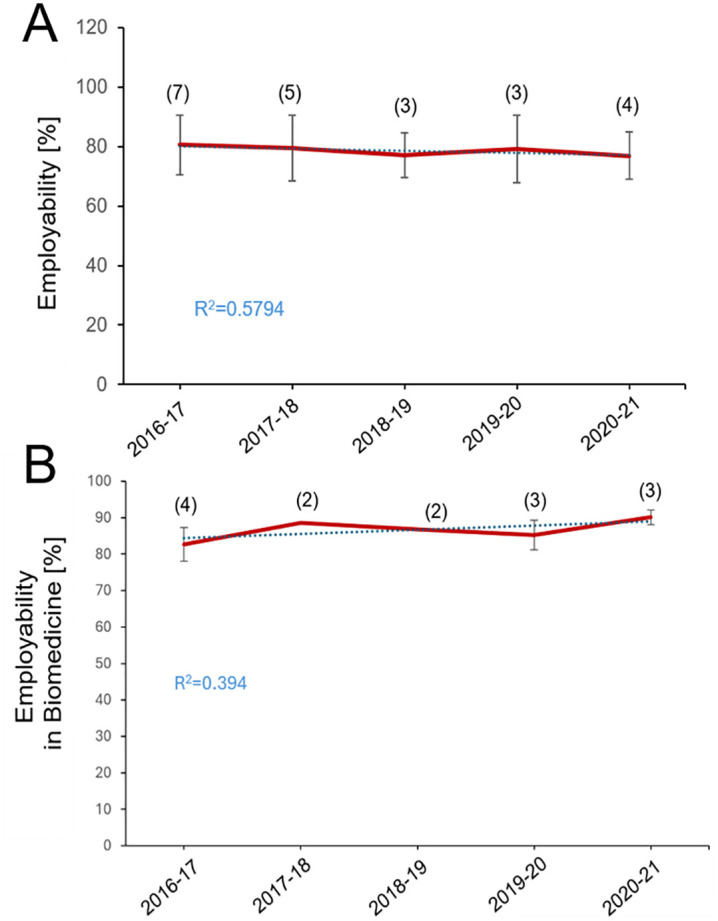
Employability rates. **A.** Data shows the mean ± SEM of the employability rates of the biomedicine graduates from 2016-17 to 2020-2021, the academic years when we have employability data. The number of Spanish universities is indicated in parentheses. Data shows employability 2-3 years after completing the degree. Data is provided by universities and national and regional agencies (AQU in Catalonia). **B.** Employability rates in biomedicine-related areas. Data shows the mean ± SEM of the employability rates in biomedicine degrees from 2016-17 to those graduated in 2021-2022. The number of Spanish universities is indicated in parentheses. Data shows employability 2-3 years after completing the degree. Data is provided by universities and regional agencies (AQU in Catalonia).

Data show that the employability rates for biomedicine degrees in Spain have been maintained over the years, with an average of the 5 academic years of 78.63%, reflecting no significant changes in the job market and the expansion of the programs across different universities (Fig 5A). The employability rate fell to 76.95% in 2020, reflecting the global job market disruption caused by the COVID-19 pandemic.

Employability (Fig 5A) includes employment in any field of graduates 2−3 years after completing their degree. Employability in Biomedicine (Fig 5B) refers to employment data related to jobs directly connected to biomedicine. The employability rates in biomedicine-related fields for graduates of biomedicine degrees in Spain (Fig 5B) have shown a generally positive trend over the years. The employability rate in biomedicine related areas increase slightly, from 82.68% in 2016−17 to 90.11% in 2020−21.

The data shown in Fig 5 are available only from 2016–17–2020–21. These data are prepared by each university and by the regional agency of Catalonia (AQU) for the Catalan universities. They are challenging to compile because they refer to employment outcomes 2–3 years after graduation.

## Discussion

Higher education in biomedicine has experienced significant growth in Spain since the degree’s introduction in the 1998−99 academic year. By 2024−25, a total of 18 universities offered specialized biomedicine programs drived by both institutional interest and a consequent increasing student demand [2,10]. This expansion correlates with a broader recognition of biomedicine’s relevance within the health sciences and its connection to global health challenges, including the COVID-19 pandemic [6]. However, the observed growth cannot be solely attributed to the pandemic, as student interest in biomedical sciences has been rising independently [11], as evidenced by the eight new programs launched since 2020–21.

Admission to these programs remains competitive. Entry marks have shown an upward trend between the 2010−11 and 2024–25 academic years. In the 2023–24 academic year, 4,614 students were enrolled across the 18 programs, confirming sustained growth in student interest. This trend suggests increasing recognition of biomedicine as a viable academic and professional path, though efforts to promote the degree and its career prospects in secondary education could further enhance visibility [12,13].

The available demographic data show a strong representation of women, with 77.1% of students in four universities identifying as female. This pattern aligns with broader trends in health-related disciplines such as medicine and pharmacy [14], and highlights biomedicine’s contribution to promoting gender balance in scientific careers.

Analysis of the curricula reveals a largely common structure among the 18 biomedicine programs, beginning with foundational training and progressing toward specialization. The first-year aims to provide a base for understanding biological systems at the molecular and cellular levels. In the second year, the courses build on foundational knowledge and introduce mechanisms relevant to human health and disease. Third-year curricula aims practical and specialized topics. At this stage, students also undertake external internships to apply their skills in real-world settings. In the final year, students complete a Final Degree Project (FDP), a research experience lasting 3–6 months, and pursue advanced coursework in different areas.

Despite a shared framework, the availability of optional subjects varies across institutions. Courses such as Biomedical Engineering, Epigenetics, Nanomedicine, Nutrition, Synthetic Biology, and Human Genetics are offered in some programs but not others, potentially leading to heterogeneity in specialized training [2].

From an educational perspective, the learning objectives, encompassing the knowledge, skills, and competencies that students are expected to attain, provides a structured framework intended to align graduate outcomes with professional requirements [15,16]. However, whether these objectives translate into preparedness for professional careers will require continued evaluation.

In 2023–24, the 18 participating universities reported a total of 115 active international agreements, primarily with European institutions. These agreements facilitate student mobility, enhance academic quality, and promote research collaboration. Participation in programs such as Erasmus+ also supports these objectives (Erasmus + , 2024).

Despite challenges, such as administrative barriers or uneven participation rates, the overall upward trend in internationalization suggest an institutional commitment to global engagement and academic cooperation.

Available data show that graduation rates in biomedicine consistently exceed national averages. In 2017–18, the graduation rate for biomedicine stood at 71.09%, compared to 53.36% across all university degrees [17]. By 2022–23, this rate had increased to 81.80%, suggesting the effectiveness of curricular design and student support mechanisms.

However, limitations remain due to the lack of publicly available and regularly updated official data [18]. The implementation of systems to identify students at risk of dropping out [19,20] and offer targeted support [21] could further enhance graduation outcomes.

Employment data indicate high levels of employability among biomedicine graduates. In 2016–17, the employability rate was 82.68%, rising to 90.11% by 2020–21 [22,23]. These outcomes suggest an alignment between academic training and labor market needs in sectors such as biomedical research, healthcare, and the pharmaceutical industry.

Although the employability rate remains high, continued monitoring is recommended to assess future trends. Recent economic indicators from Spain and the EU [24] are favorable, which may support sustained or improved employment outcomes for graduates.

Despite the strengths of this study, a key limitation is the lack of qualitative data from students, academics, and industry representatives, which restricts contextual understanding of the quantitative trends. Incorporating interviews, surveys, or focus groups in future research would provide deeper insight into motivations, satisfaction, and the alignment between academic preparation and labor market needs.

## Conclusions

The findings of this study offer a comprehensive overview of biomedicine education in Spain. These results may inform educational policy and institutional strategies, and contribute to broader discussions on interdisciplinary training and workforce development in the biomedical sciences.

## Supporting information

S1 Table List of Spanish universities offering a degree in Biomedicine, including the official program name and links to each university’s website. (XLSX)

S2 Table The complete study dataset (1998–2023), compiling structural, academic, and outcome-related variables such as program availability, student enrollment, admission criteria, curricular structure, international collaborations, graduation rates, and employment outcomes across the 18 universities analyzed. (XLSX)
